# Ten years of tuberculosis intervention in Greenland – has it prevented cases of childhood tuberculosis?

**DOI:** 10.3402/ijch.v73.24843

**Published:** 2014-07-11

**Authors:** Emilie Birch, Mikael Andersson, Anders Koch, Flemming Stenz, Bolette Søborg

**Affiliations:** 1 Faculty of Health Sciences, University of Copenhagen, Copenhagen, Denmark; 2 Department of Epidemiology Research, Statens Serum Institut, Copenhagen, Denmark; 3 The National Board of Health, Nuuk, Greenland

**Keywords:** epidemiological study, tuberculosis disease, tuberculosis control, children, Greenland

## Abstract

**Background:**

The incidence of tuberculosis (TB) disease in Greenland doubled in the 1990s. To combat the increase, national TB interventions were initiated in 2000 and strengthened in 2007.

**Objective:**

To determine whether the effect of interventions could be detected, we estimated the TB disease risk among children≤15 years before and after interventions were implemented.

**Design:**

For a study cohort, we recruited all children ≤15 years of age included in the Greenlandic Civil Registration System (CRS) from 1990 to 2010. The CRS identifier was used to link cohort participants with TB cases identified based on the Greenlandic National TB registry. Bacille Calmette Guerin (BCG) vaccination status was identified through year of birth, as BCG was offered to newborns born either before 1991 or after 1996. Years with interventions were defined as 2000–2006 (primary interventions) and 2007–2010 (intensified interventions). Risk of TB was estimated using Poisson regression.

**Results:**

The study included 35,858 children, of whom 209 had TB disease. The TB disease incidence decreased after interventions were implemented (2007–2010: IRR [incidence rate ratios] 0.62, 95% CI: 0.39–0.95, p=0.03, compared with the 1995–1999 period). The TB disease risk was inversely associated with BCG vaccination (IRR: 0.54, 95% CI: 0.41–0.72, p<0.001).

**Conclusions:**

Years with national TB interventions in Greenland, including neonate BCG vaccination, are associated with a lower TB disease incidence among children ≤15 years of age.

In the 1950s, the Inuit population in Greenland presented the highest tuberculosis (TB) disease incidence rates (IR) ever observed worldwide with an IR of 2,200 cases per 100,000 inhabitants in 1952 (1). Subsequently, TB interventions were initiated including inauguration of Dronning Ingrids Sanatorium (SANA) in Nuuk, Greenland, in 1954, a hospital with 211 beds exclusively for treating TB patients. From 1955 to 1971, a specialized TB ship sailed the coastline offering bi-annual population-based chest x-ray screenings. The Bacille Calmette Guerin Vaccination (BCG) was introduced in 1949 and became a nationwide offer for neonates from 1955 (2). The collaborative interventions dramatically reduced the TB disease IR, which came to an all-time low of 25 cases per 100,000 inhabitants in 1983 (3).

With the declining incidence of TB disease, the Greenlandic TB interventions were phased out and the last remaining intervention, the BCG vaccine to neonates, was removed from the vaccination programme in 1991. However, from the 1990s, the TB disease IR increased again (3). In addition to a doubling of the overall TB incidence, numerous cases of TB meningitis among young children with fatal outcomes were registered. These fatal cases led to the reintroduction of BCG vaccination offered to all newborns in 1997.

In the year 2000, the Greenland Home Rule Government that was formed in 1979 introduced its first national TB intervention programme in response to the increased TB disease incidence in the 1990s. TB interventions in the 1950s were instituted by the Danish Government. The national TB intervention programme in 2000 included the formation of a central TB group to coordinate the national interventions.

National guidelines were formed describing procedures for diagnosis, treatment, contact tracing and screening. A national TB nurse was recruited for countrywide coordination and training of staff (3). A central monitoring unit collecting TB notifications from all districts was established (4). In the early 2000s, a study documented that 8% of Greenlandic school children were infected with *Mycobacterium tuberculosis* infection (MTI) (5). The study results led to the implementation of an intensified intervention strategy in 2007. The additional interventions included yearly MTI screening of school children, public TB campaigns and publication of TB training material for health workers (6,7).

The purpose of the present study was to determine whether 10 years of national TB interventions are associated with any change in childhood TB disease incidence in Greenland.

## Materials and methods

### Study design and population

A retrospective cohort study was conducted. The study population consisted of all children living in Greenland under the age of 16 years in the period 1 January 1990 to 31 December 2010. Children born in this period were included and children turning 16 were removed.

At birth, all Greenlandic citizens are registered in the Danish Civil Registration System (CRS) and allocated a personal identifier, which allows follow-up in all public registries. Information on age, sex and place of birth of participants was collected from the CRS identifier. Only children born in Greenland were included. Follow-up time was contributed until; TB notification, emigration from Greenland or Denmark, or until the end of the study period, whichever came first.

TB is a mandatory notifiable disease in Greenland and is reported to the National Board of Health in Greenland. Cases are reported on an individual level using the CRS identifier and data are stored in the TB registry. Using the personal CRS identifier, all TB cases among study participants were sought in the Greenlandic TB registry for the period 1990–2010.

In Greenland, the TB disease diagnosis is made based on clinical criteria combined with laboratory analysis, such as positive *Mycobacterium tuberculosis (Mtb)* microscopy and/or culture. Mantoux test or QuantiFERON^®^-TB Gold test may be done as part of the evaluation (8). The diagnosis is, however, not easy in children because they may not have symptoms that are more typically seen in adults, such as cough (9). The diagnosis is often made clinically without a positive *Mtb* microscopy. Bacteria in sputum as well as x-ray changes are often difficult to detect in children, but are easier to detect when the children are older at the time of diagnosis (10).

BCG vaccination status for each child was determined by the year of birth. The official BCG vaccination programme in Greenland mandated vaccination of all newborns born either before 1991 or after 1996 (2). Children born from 1991 to 1996 are therefore considered not to be vaccinated, while children born in 1990 and 1997–2010 are considered to be BCG vaccinated.

### Statistical methods

To determine whether TB disease incidence was associated with TB interventions, we calculated TB disease incidence in the cohort of children in sub-periods from 1990 to 2010, where 1990–1999 was the pre-intervention period, and 2000–2010 the intervention period. As interventions were intensified in 2007, the intervention period was sub-divided into the periods – 2000–2006 and 2007–2010. The pre-intervention period was correspondingly divided into the 2 periods – 1990–1994 and 1995–1999. The period 1995–1999 was used as reference due to the high TB disease incidence in this period.

All included children contribute person years individually using the entry and exit dates as described. IR were calculated as the number of cases divided with the number of observed person years scaled appropriately.

The incidence rate ratios (IRRs) were calculated using log-linear Poisson regression models adjusted for age, sex and BCG status as the yearly number of new TB cases by year of follow-up. The age adjustment was made with cubic splines restricted to be linear in the tails (11). SAS PROC Genmod was used (SAS version 9.3, SAS Institute, Cary, NC, USA).

Age, sex and BCG vaccination status were tested together with the periods in a multivariable regression analysis, since these factors could influence TB outcome.

We not only adjusted the multivariable model with age using splines but also showed the adjusted effects of age in groups not using splines. Further, we also performed a trend test for the age effect (age in years).

### Ethical considerations

The studies fulfilled the Helsinki Declaration II. The Commission for Scientific Research in Greenland approved the studies (Approval No. 2014-6). Data were stored in accordance with the regulation of the Danish Data Protection Agency. The studies were carried out in accordance with the STROBE statements.

## Results

The study included 35,858 children, hereof 51% males. In the study period from January 1990 to December 2010, the total number of TB cases among children younger than 16 were 209, of whom 87 (42%) were notified in the period 1990–1999, 93 (44%) in the period 2000–2006 and 23 (14%) from 2007 to 2010.

IRRs with confidence intervals and p-values for the 4 different periods are given in Table I. The IRRs were significantly lower in the first pre-intervention period 1990–1994 and in the second post-intervention period 2007–2010 compared with the periods immediately before (1995–1999) and after initiation of interventions (2000–2006), which showed no difference in incidence.

**Table I:** TB disease among children in Greenland, 1990 to 2010, stratified by period

Period^a^	TB cases	IRR^b^	95% CI^c^	p^d^	IR^e^
1990–1994	23	0.47	(0.28–0.75)	0.002	29.0 (18.7,42.5)
1995–1999	64	1 (Ref.)	–	–	75.8 (58.7,95.9)
2000–2006	93	1.05	(0.76–1.45)	0.78	81.6 (66.1,99.3)
2007–2010	29	0.62	(0.39–0.95)	0.03	49.0 (33.3,69.1)
Total	209			0.0004	

a Adjusted for sex, vaccination period and age with splines.

b Incidence rate ratios.

c Confidence intervals.

d Test for homogeneity.

e Incidence rates per 100,000 person years.

Table II illustrates that the prevalence of TB disease was highest among girls with an IRR of 1.37, P=0.03. BCG vaccination in the neonatal vaccination programme was associated with a lower TB IRR (0.54, P<0.001).

**Table II:** Factors associated with TB disease among Greenlandic children, 1990–2010

	TB cases	IRR (95% CI)	p
Total	209		
Sex^a^			
Girls	119	1.37 (1.04–1.80)	0.0250
Boys	90	1	
Born in BCG vaccination period^a^			
Yes	111	0.54 (0.41–0.72)	<0.0001
No	98	1	
Age^b^			
0 years	10	1	
1–5 years	59	1.08 (0.58–2.24)	0.8253
6–10 yeas	64	1.11 (0.59–2.30)	0.7632
11–15 years	76	1.42 (0.77–2.93)	0.3045
Age trend^b^		1.04 (1.01–1.07)	0.02

a Adjusted for period, sex, vaccination period and age with splines.

b Adjusted for period, sex and vaccination period.

### Age trend in risk of TB

There was an adjusted linear trend for age with an IRR of 1.04, P=0.02.

## Discussion

### Impact of TB interventions

This study reports that the increasing incidence in TB disease among children in Greenland seen through the 1990s came to a halt in the period 2000–2006 and decreased from 2007. This coincides with the introduction of interventions against TB in 2000 and the strengthening of the programme from 2007. Obviously, it is difficult to claim a causal association between TB interventions and TB disease incidence based on temporal associations, but it is striking that the epidemic stopped during the first period of interventions and that the incidence decreased during the second. An actual decrease in incidence immediately following initiation of TB interventions in 2000 would not have been likely, as the effort to control TB is a long-term effort (5,12). Therefore, while it seems reasonable to assume that TB interventions have had an impact on TB disease incidence in Greenland, it is difficult to determine whether the decrease in incidence after 2007 is the result of strengthening interventions or a delayed effect of the initial interventions in 2000. For the same reason, it is difficult to identify the contribution of individual elements of the intervention programmes.

Three factors were significantly associated with increased risk of TB disease in our population – female sex, increasing age and being born in a period without neonatal BCG vaccination (1991–1996). While sex and age distributions were not affected by the intervention programmes, the reintroduction of the BCG vaccination may have played a role in the reduced TB incidence after 2000. Neonatal BCG vaccination was stopped in 1991 in Greenland and reintroduced in 1997. Worldwide, the effect of the BCG vaccination is much debated and vaccination has shown variable efficacy against TB in different settings (13,14). However, there is evidence that BCG has an effect in preventing disseminated TB disease in young children (15,16). It is possible that the reintroduction of the neonatal BCG vaccination may, with or without the other TB interventions from 2000, have played a role in the prevention of TB in Greenland.

Greenlandic girls had a significantly higher risk of TB disease compared with Greenlandic boys. This finding contrasts with the generally well-known sex distribution for TB disease among adults according to which men have a higher risk of TB disease than women (17). The higher TB risk among men is not strictly a sex-effect but rather presumed to be related to certain social factors found in higher frequencies in men, for example, alcohol abuse (18). New DNA-typing-based studies report that TB transmission most often happens within the household (19). A possible explanation of our study findings of a higher risk among Greenlandic girls could be that Greenlandic girls spend more time inside the house, leaving them more exposed to MTI from TB-infected members of the household.

The risk of TB disease increased with age by 4% per year, reflecting increased risk of TB disease with increasing period of exposure. The figure of 4% is high. The figure of 4% covers the whole period 1990–2010 and is obviously dependent on time.

We previously found an annual risk of MTI (i.e. exposure to Mtb measured by Mantoux test or QuantiFERON^®^-TB Gold test) of 0.8% per year in children in Greenland in the period 2006–2007 (5).

### Strengths and limitations

Data were gathered from national registers. The population is well-described with every citizen of Greenland being registered with vital information in the weekly updated person-identifiable national CRS. Also, all Greenlandic children have the possibility to appear in the National Greenlandic TB registry, as it holds nationwide information on all known cases of TB disease in Greenland. However, it cannot be ascertained that all cases of TB disease in Greenland are diagnosed. Like every other disease, a TB diagnosis depends on the patients seeking medical help, doctors’ diagnoses and reporting to the system and possible contact tracing. However, while underdiagnosis of the TB disease might have been the case in the 1990s, possibly suggested by the observation of very variable risks of transmission in Greenland (20), the increased attention to TB both from the medical system and the public as well as the contact tracing make underreporting of TB disease less likely after 2000. If increased awareness had led to more cases being recognized after 2000 this would have led to a higher incidence after this time. Therefore, the actual reduction in incidence seen after 2000 indicates a true decrease in risk following introduction of interventions.

### TB in Greenland

Greenland's healthcare system is comparable with those of other Western countries. The population is well-defined and despite scattered settlements it is possible to organize focused preventing health interventions. Despite this fact, the TB disease incidence is similar to that in developing countries. The explanation is probably multi-factorial. One reason could be that the cold climate in Greenland favours indoor activities, leading to a higher risk of MTI through longer lifespan of bacteria indoors and easier Mtb transmission through less ventilated houses. Another reason could be the Inuit tradition of large families living in small households with poor sanitary standards. A debated, but unsolved, issue is whether genetic factors predispose the Inuit population to higher risks of TB disease (5).

Further efforts are needed to reduce the TB disease incidence in Greenlandic children. Experts from WHO visited Greenland in 2010 to evaluate the TB situation and found an unacceptably high incidence of TB disease (21). Thus, TB interventions from 2000 do not seem sufficient to fulfil the ambitions of WHO to reduce the incidence of TB disease by 50% by 2015 compared with 1990 (22). For this reason, a new intervention programme, “National TB strategy 2012–2016,” has been implemented (8). This programme focuses especially on information campaigns, diagnostics and treatment in the local environment with employment of local TB nurses and TB coordinators.

Time will show if the new intervention is capable of further controlling and reducing TB disease among children in Greenland. It has previously been possible to control TB in Greenland and to achieve impressive results within a few decades. Replication of such achievements should be possible by nationwide campaigns.

## Conclusions

This study indicates that the first 10 years of Greenlandic National TB interventions have had a positive impact on TB disease in Greenlandic children with significantly lower IRs after initiation of interventions. While the increasing incidence seen throughout the 1990s came to a halt following the first interventions in 2000, the incidence was reduced after a strengthening of interventions in 2007.
